# Sputum mediator profiling and relationship to airway wall geometry imaging in severe asthma

**DOI:** 10.1186/1465-9921-14-17

**Published:** 2013-02-11

**Authors:** Dhananjay Desai, Sumit Gupta, Salman Siddiqui, Amisha Singapuri, William Monteiro, James Entwisle, Sudha Visvanathan, Harsukh Parmar, Radhika Kajekar, Christopher E Brightling

**Affiliations:** 1 Institute for Lung Health, NIHR Respiratory Biomedical Research Unit, Department of Infection, Immunity & Inflammation, University of Leicester, Leicester, UK; 2 Department of Respiratory Medicine University Hospitals of Leicester NHS Trust, Leicester, UK; 3 Department of Radiology, Wellington Hospital, Leicester, New Zealand; 4 Department of experimental Medicine, Hoffmann-La Roche, Nutley, NJ, USA

**Keywords:** Asthma, Remodelling, RB1 bronchus

## Abstract

**Background:**

Severe asthma is a heterogeneous disease and the relationship between airway inflammation and airway remodelling is poorly understood. We sought to define sputum mediator profiles in severe asthmatics categorised by CT-determined airway geometry and sputum differential cell counts.

**Methods:**

In a single centre cross-sectional observational study we recruited 59 subjects with severe asthma that underwent sputum induction and thoracic CT. Quantitative CT analysis of the apical segment of the right upper lobe (RB1) was performed. Forty-one mediators in sputum samples were measured of which 21 mediators that were assessable in >50% of samples were included in the analyses.

**Results:**

Independent of airway geometry, sputum MMP9 and IL-1β were elevated in those groups with a high sputum neutrophil count while sputum ICAM was elevated in those subjects with a low sputum neutrophil count. In contrast, sputum CCL11, IL-1α and fibrinogen were different in groups stratified by both sputum neutrophil count and airway geometry. Sputum CCL11 concentration was elevated in subjects with a low sputum neutrophil count and high luminal and total RB1 area, whereas sputum IL1α was increased in subjects with a high sputum neutrophil count and low total RB1 area. Sputum fibrinogen was elevated in those subjects with RB1 luminal narrowing and in those subjects with neutrophilic inflammation without luminal narrowing.

**Conclusions:**

We have demonstrated that sputum mediator profiling reveals a number of associations with airway geometry. Whether these findings reflect important biological phenotypes that might inform stratified medicine approaches requires further investigation.

## Introduction

Asthma affects up to 5% of the adult population and approximately 10% of asthmatics have severe or refractory disease [1,2]. This group represents an unmet need as they consume a disproportionate amount of healthcare resources, and contribute to the mortality and morbidity of the disease. Severe asthma is a complex disease with phenotypic heterogeneity in terms of the pattern and intensity of airway inflammation and remodelling and clinical expression of disease [3]. Understanding the underlying pathobiology of severe asthma phenotypes mainly those with Th2 high [4] or eosinophilic airway inflammation [5] has translated into recent success with anti IL-5 [6] and anti IL-13 therapy [7,8]. In particular, anti-IL5 led to improvements in exacerbation frequency and airway remodelling in terms of sub-epithelial matrix deposition and airway wall geometry determined by CT analysis [6]. Understanding the relationship between the inflammatory profile in the airway and airway geometry may help to identify patient phenotypes that may be more amenable to strategies to modulate airway remodelling and might prioritise potential targets within stratified populations for specific highly targeted therapies.

We and others have used quantitative and qualitative CT image analysis to define the airway geometry in asthma [9-11]. In a previous report, we found that airway wall remodelling defined by CT was most closely associated with neutrophilic inflammation and airflow obstruction but was poorly associated with patient reported outcomes [10]. This association between persistent neutrophilic inflammation and lung function decline is a consistent observation in asthma and COPD [12,13]. Sputum and bronchoalveolar lavage cytokine profiling by analyses of protein and transcriptomics in asthma and chronic obstructive pulmonary disease has demonstrated that patterns of inflammatory mediators are associated with cellular profiles and clinical outcomes [14-17]. We hypothesised that there is differential sputum mediator profiles in severe asthmatics stratified by airway geometry determined by CT analysis. To test our hypothesis we measured sputum mediators in patients that had undergone CT analysis and i) dichotomised subjects by the median right upper lobe apical bronchus (RB1) luminal area or the median RB1 total area and ii) stratified by the median sputum neutrophil count and airway geometry.

## Methods

### Subjects

We performed a single centre retrospective cross-sectional study based upon the difficult asthma clinic (DAC) at Glenfield Hospital, Leicester, UK. Fifty-nine subjects out of the 92 included in a previous quantitative CT analysis between 2004 to 2008 were included in this study if they had adequate sputum samples for mediator analysis (>150 μL supernatant volume). Sputum was obtained at the routine clinical visit immediately before or after the CT scan. All assessments were undertaken at stable visits at least 2 months after a severe exacerbation.

Subjects attending DAC undergo an extensive re-evaluation, as part of their routine clinical care including an extensive history, skin prick tests for common aeroallergens, spirometry, methacholine challenge tests, sputum induction [18] and asthma control questionnaire (ACQ_6_) [19]. A history of severe exacerbations was defined as worsening of symptoms requiring ≥3 days of high dose systemic corticosteroids [20]. The diagnosis of asthma is confirmed by a respiratory physician based on history and one or more of the following objective criteria (maximum diurnal peak expiratory flow variability >20% over a 2 week period, significant bronchodilator reversibility defined as an increase in FEV_1_ of >200mls post bronchodilator or a PC_20_ methacholine of <8 mg/ml). Severe asthma was defined in accordance with the American Thoracic Society (ATS) workshop on refractory asthma [21]. Informed consent for clinical characterisation and computed tomography was obtained from all subjects and the study was approved by the Leicestershire, Northamptonshire and Rutland Research Ethics Committee.

### Cross sectional imaging

HRCT was performed using Siemens Sensation 16 scanner. Scans were acquired using standard HRCT protocol (sequential scanning at 10-mm increments with 1-mm collimation) from the apex of the lung to the diaphragm [10]. Patients were scanned in the supine position at maximal inspiration (adequate breath holding rehearsed prior to scan), with their arms held over their heads. Images were reconstructed using a high spatial frequency algorithm, through a 512X512 matrix, with a small field of view targeted to image only pulmonary areas. Scanning time ranged from 30-45 s with a voltage of 120kVp and peak effective tube current (dose modulation based on size and attenuation profile of the region scanned used to minimise radiation dose) of 140 mAs (range 65-140 mAs).

### Qualitative and quantitative airway analysis

CT scans were analysed qualitatively by a single observer and the presence or absence of bronchiectasis and/or bronchial wall thickening was reported as described previously [9]. An automated program Emphylyx-J V 1.00.01 [22,23] using the full-width at half-maximum (FWHM) technique was used to determine the airway cross-sectional geometry. Image data were transferred from the CT workstation to a personal computer in DICOM 3.0 format. After identifying the RB1 the operator placed a seed point in the airway lumen from which 64–128 rays were cast across the airway wall. The boundaries of the wall were defined by the midpoint of the profile of CT numbers across each ray. Lumen Area (LA), wall area (WA), maximum and minimum airway diameter were measured. High spatial frequency algorithm was used for reconstruction of images as it results in reduced blurring and, as demonstrated by phantom studies, [24] is associated with reduced errors in airway wall estimation using FWHM method. Correction equations for both size dependant error using the FWHM method and oblique orientation of airways were applied as previously described [10] LA and WA were corrected for body surface area (BSA). Total area (TA) and percentage WA (%WA) were derived from LA and WA (TA = LA + WA; %WA = WA/TA x100).

### Sputum processing and cytokine assessment

The sputum was selected, dispersed using mucolytic dithiothreitol, and processed to generate a sputum differential cell count and cell-free supernatants [25,26] Measurements for validated mediators were measured using multiplex assays from Myraid RBM (Austin, TX, USA). We included mediators that have been previously validated in sputum and were measurable in >50% of samples. The full list of mediators is as shown Table 1.

**Table 1:** Mediators measured in sputum samples

**Mediators included in analysis**	**Not measurable in >50 % of samples, excluded**
Factor VII	Brain-Derived Neurotrophic Factor (BDNF)
Fibrinogen	C-Reactive Protein (CRP)
Ferritin (FRTN)	Granulocyte-Macrophage Colony-Stimulating Factor (GM-CSF)
Intercellular Adhesion Molecule 1 (ICAM-1)	Interferon gamma (IFN-gamma)
Interleukin-1 alpha (IL-1 alpha)	Interleukin-17 (IL-17)
Interleukin-1 beta (IL-1 beta)	Interleukin-2 (IL-2)
Interleukin-1 receptor antagonist (IL-1ra)	Interleukin-3 (IL-3)
Interleukin-6 (IL-6)	Interleukin-4 (IL-4)
Interleukin-8 (IL-8)	Interleukin-5 (IL-5)
Interleukin-15 (IL-15)	Interleukin-7 (IL-7)
Interleukin-18 (IL-18)	Interleukin-10 (IL-10)
Monocyte Chemotactic Protein 1 (CCL2)	Interleukin-12 Subunit p70 (IL-12p70)
Macrophage Inflammatory Protein-1 beta (CCL4)	Interleukin-12 Subunit p40 (IL-12p40)
Eotaxin-1 (CCL11)	Interleukin-23 (IL-23)
Matrix Metalloproteinase-3 (MMP-3)	Macrophage Inflammatory Protein-1 alpha (CCL-3)
Matrix Metalloproteinase-9 (MMP-9)	Matrix Metalloproteinase-2 (MMP-2)
Tissue Inhibitor of Metalloproteinase 1 (TIMP-1)	Stem Cell Factor (SCF)
Tumor Necrosis Factor alpha (TNF-alpha)	T-Cell-Specific Protein (CCL-5)
Tumor Necrosis Factor Receptor 2 (TNFR2)	Tumor Necrosis Factor β (TNF-β)
Vascular Cell Adhesion Molecule-1 (VCAM-1)	von Willebrand Factor (vWF)
Vascular Endothelial Growth Factor (VEGF)	

### Statistical analysis

Statistical analysis was performed using PRISM version 4 (GraphPad Software, San Diego, CA), SAS version 8.02 (SAS Institute Inc. Cary, NC) and SPSS version 16 (SPSS, Inc. Chicago, IL). Parametric and non-parametric data are presented as mean (standard error of the mean) and median (interquartile range) respectively. Subjects were dichotomised into high and low luminal area, total area and wall area using the median values as the cut-offs (RB1 luminal area LA/BSA (≤5 mm^2^/m^2^ versus >5 mm^2^/m^2^) or total area TA/BSA (≤16 mm^2^/m^2^ versus >16 mm^2^/m^2^) or wall area WA/BSA (≤11.5 mm^2^/m^2^ versus >11.5 mm^2^/m^2^). Between group differences were analysed by unpaired t-tests and Mann–Whitney tests as appropriate. Spearman correlation coefficient was used to determine the relationship between RB1 dimensions (LA/BSA, WA/BSA, TA/BSA and %WA) and clinical indices and sputum mediator data. Our previous study highlighted the relationship between CT-determined remodelling and neutrophilic inflammation [10]. Therefore, we further stratified the population into four groups using the medians as cut-offs for the differential sputum neutrophil counts (≤66% versus >66%) and the RB1 total area or luminal area. Comparisons across groups were made by Kruskal-Wallis test with post hoc Dunn’s pair-wise comparisons. A p value of <0.05 was taken as statistically significant.

## Results

**Figure 1:**
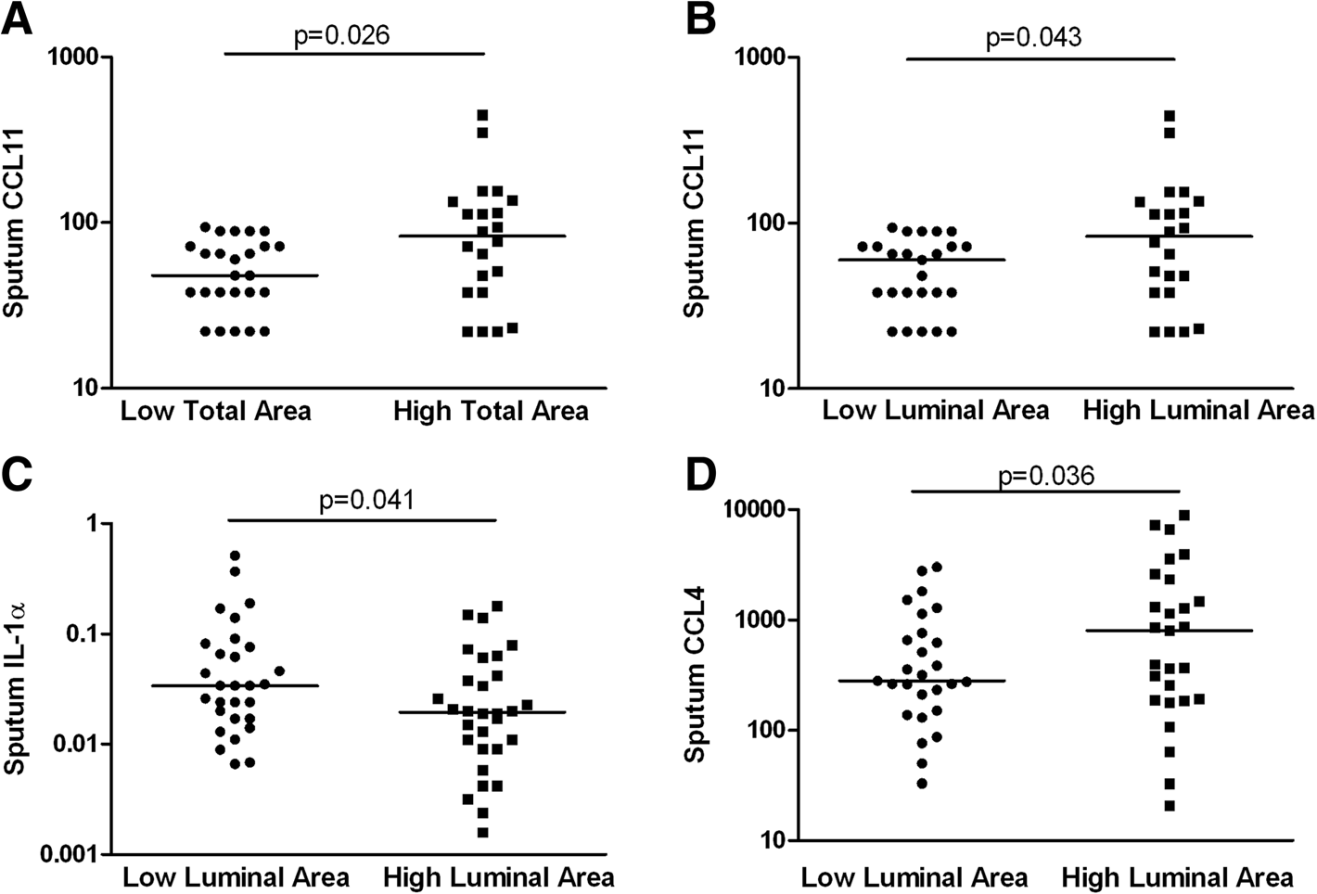
**A) Subjects stratified by low or high RB1 total area on the X axis, their corresponding sputum CCL11 levels (pg/ml) on the log transformed Y axis.** Bar represents median value, p value by Mann Whitney *U* test, and subjects stratified by low or high RB1 luminal area on the X axis, their corresponding **B**) sputum CCL11 (pg/ml), **C**) sputum IL-1α (pg/ml) and **D**) sputum CCL4 (pg/ml) on the log transformed Y axis. Bar represents median value, p value by Mann Whitney *U* test.

The clinical and CT imaging characteristics and sputum mediator concentrations for the whole population and dichotomised by the median RB1 total and luminal areas are as shown (Tables 2 and 3). Subjects with a low total area and luminal area were older with a later age of onset of disease, higher body mass index, more bronchiectasis and more frequent exacerbations (Table 2). There was marked heterogeneity in mediator concentrations within and between the groups of subjects and only the sputum CCL11 concentration was significantly different in the groups stratified by median RB1 total area ([48.3 versus 76.3 pg/ml], p = 0.026). Further differences were observed between subjects stratified by RB1 luminal area (Figure 1, Table 3) for sputum CCL11 ([49.1 versus 74.9 pg/ml], p = 0.043), IL-1α ([38 versus 19 pg/ml], p = 0.043) and CCL4 ([349 versus 611 pg/ml] p = 0.036). RB1 luminal area correlated with sputum CCL11 (r = 0.31, p = 0.03) but there were no significant correlations between RB1 total or luminal area and other mediators. There were no differences in cytokine concentrations between those with high or low wall area.

**Table 2:** Clinical features of subjects stratified by the median RB1 total area and luminal area

	**Total**	**Low total area**	**High total area**	**p value**	**Low luminal area**	**High luminal area**	**p value**
N	59	30	29		30	29	
Male, n	25	12	13	0.37	12	13	0.37
Current age	49 (2)	53 (2)	45 (2)	**0.02**	53 (2)	45 (2)	**0.024**
Age of onset	28 (2.5)	34 (3.5)	21 (3)	**0.037**	32(3.5)	22 (3)	**0.041**
BMI, kg/m^2^	29.8 (0.8)	32.3 (1.2)	27.4(1.1)	**0.003**	32.6 (1.1)	27.1 (1.1)	**0.001**
Habitual smokers and ex smokers with >10 py	13	10	3	0.16	9	4	0.3
Pack years history	8 (2.5)	11 (5)	6 (2.5)	0.36	11 (5)	6 (2.5)	0.23
Severe Exacerbations	2	2 (0.3)	3 (0.4)	**0.03**	2 (0.3)	3 (0.4)	**0.03**
IgE	153 [97–241]	145 [74–183]	162 [84–313]	0.87	143 [82–325]	167 [76–270]	0.89
ACQ_6_	2.18 (0.13)	2.2 (0.17)	2.14 (0.23)	0.74	2.21 (0.17)	2.15 (0.23)	0.76
Inhaled Corticosteroid dose	2000 [400–4000]	2000 [400–4000]	2000 [640–4000]	0.28	2000 [400–4000]	2000 [640–4000]	0.43
Subjects taking oral corticosteroids	25	15	10	0.72	15	10	0.72
Daily prednisolone dose, mg	4.5 (0.7)	6 (1.2)	2.5 (0.7)	0.54	5.5 (1.5)	3.5 (0.8)	0.83
Pre Bronchodilator FEV_1_	2.26 (0.12)	2.24 (0.17)	2.2 (0.16)	0.9	2.18 (0.17)	2.34 (0.15)	0.49
Pre Bronchodilator FEV_1_% predicted	2.42 (0.12)	2.4 (0.18)	2.4 (0.16)	0.98	2.33 (0.18)	2.5 (0.15)	0.48
BD reversibility, ml	0.23 (0.07)	0.31 (0.14)	0.13 (0.03)	0.23	0.29 (0.13)	0.15 (0.03)	0.36
Pre Bronchodilator FVC	3.26 (0.1)	3.22 (0.21)	3.29 (0.20)	0.87	3.1 (0.22)	3.3 (0.18)	0.39
Pre Bronchodilator FVC	3.35 (0.1)	3.36 (0.21)	3.32 (0.20)	0.88	3.27 (0.21)	3.42 (0.18)	0.61
Pre Bronchodilator FEV_1_% of predicted	75.2 (3)	76 (4)	74.3 (3.6)	0.79	74.3 (5)	76 (3.4)	0.77
Post Bronchodilator FEV_1_% of predicted	80.8 (2.9)	80.8 (4)	80.8 (3.4)	0.99	78.4 (5)	83.1(3.2)	0.42
Bronchodilator Reversibility %FEV1	5.9 (0.97)	5.4 (1.4)	6.4 (1.7)	0.58	4.7 (0.8)	7.1 (1.7)	0.22
Pre Bronchodilator FEV_1_/FVC ratio	69.5 (1.8)	69 (2.6)	69.3 (2.7)	0.82	69.6 (2.4	69.4 (2.5)	0.95
Post Bronchodilator FEV_1_/FVC ratio	72.1 (1.7)	78 (2.5)	72.8 (2.5)	0.62	70.7 (2.4)	73.4 (2.4)	0.44
Total Cells	0.5 [0.4-0.8]	0.6 [0.3-1.1]	0.5 [0.3-0.8]	0.61	0.6 [0.3-1]	0.5 [0.3-0.8]	0.77
Neutrophils %	58.3 [48–69.5]	55.6 [40.7-76.3]	60 [50.1-72.6]	0.97	56.2 [47–77]	59.9 [49.5-72.5]	0.76
Eosinophils %	2.2 [1.5-3.4]	2.5 [1.3-4.5]	2 [1.1-3.7]	0.43	2.2 [1.1-4.2]	2.2 [1.2-4]	0.80
Bronchiectasis present,%	23 (39)	7(30)	16 (69)	**0.02**	9 (39)	14 (60)	0.16
Bronchial Wall Thickening present,%	39 (67)	21(53)	18 (46)	0.66	22 (56)	17 (43)	0.22
Lumen area/Body Surface Area (mm^2^/m^2^)	5.3 (0.3)	3.4 (0.2)	7.2 (0.42)	**0.001**	3.2 (0.2)	7.3 (0.4)	**0.001**
Total area/Body Surface Area (mm^2^/m^2^)	17.8 (0.8)	12.7 (0.6)	22.8 (1)	**0.001**	13.2 (0.8)	22.3 (0.9)	**0.001**

**Table 3:** Sputum mediator concentrations for subjects stratified by the median RB1 total area and luminal area

	**Total**	**Low total area**	**High total area**	**p value**	**Low luminal area**	**High luminal area**	**p value**
Factor VII*	2.15 [1.89-2.43]	2.03 [1.69-2.44]	2.27 [1.89-2.72]	0.40	2.01 [1.68-2.41]	2.30 [1.91-2.77]	0.25
Fibrinogen*	240 [172–335]	260 [161–421]	223 [137–364]	0.48	291 [184–461]	200 [121–330]	0.16
Ferritin*	2.21 [1.54-3.17]	2.56 [1.62-4.06]	1.94 [1.10-3.42]	0.38	2.21 [1.35-3.61]	2.21 [1.27-3.85]	0.9
IL-1 α	27 [19-37]	35 [23–54]	20 [12-33]	0.13	38 [25–58]	19 [12-30]	**0.041**
IL-1 β	47 [31–71]	44 [26–76]	49 [25–96]	0.40	45.4 [26.72-77.14]	48.8 [25–95.4]	0.40
IL-1RA	6951 [5168–9350]	8147 [5120–12964]	5962 [4033–8815]	0.59	9108 [5752–14421]	5353 [3662–7824]	0.19
IL-6	41 [30–56]	32 [20–53]	52 [35–76]	0.14	33.3 [20.5-54.1]	51.8 [35.3-76.1]	0.16
IL-8	3608 [2462–5287]	2846 [1599–5064]	4537 [2686–7665]	0.37	3062 [1790–5237]	4227 [2393–7469]	0.43
IL-15	440 [370–520]	410 [320–540]	460 [360–600]	0.60	410 [320–540]	460 [360–600]	0.60
IL-18	40.2 [32.2-50.2]	37.2 [27–51.3]	43.3 [31.2-6]	0.31	39.2 [27.7-55.5]	41.2 [30.5-55.6]	0.53
TNF-α	6.46 [4.58-9.12]	4.66 [3.45-7.30]	9.35 [3.91-17.8]	0.09	4.71 [3.48-6.36]	9.25 [4.84-17.6]	0.06
TNFR2	330 [230–490]	280 [160–510]	390 [220–670]	0.46	290 [160–500]	390 [220–680]	0.39
CCL2	100 [75–134]	96 [62–150]	105 [71–156]	0.35	100 [65–153]	101 [67–153]	0.98
CCL4	462 [313–682]	380 [241–600]	554 [291–1052]	0.42	349 [220–555]	611 [322–1158]	**0.036**
CCL11	59.8 [48.4-73.9]	48.3 [39.2-59.6]	76.3 [52.4-111]	**0.026**	49.1 [39.7-60.7]	74.9 [51.3-109]	**0.043**
MMP-3*	0.25 [0.19-0.33]	0.27 [0.18-0.40]	0.23 [0.16-0.33]	0.51	0.26 [0.18-0.39]	0.24 [0.16-0.34]	0.56
MMP-9*	38.1 [26.7-54.3]	48.8 [29.1-81.8]	30.3 [18.3-50]	0.11	50.8 [31.6-81.7]	29.1 [17.1-49.5]	0.09
TIMP-1*	108 [78.6-14]	93 [56–155]	124 [82–188]	0.33	98 [60–159]	118 [75–185]	0.40
ICAM-1	2900 [2170–3870]	2490 [1650–3740]	3370 [2200–5150]	0.32	2400 [1620–3550]	3530 [2280–5460]	0.19
VCAM-1	1130 [850–1490]	1240 [830–1860]	1003 [690–1560]	0.80	1200 [820–1760]	1060 [690–1630]	0.78
VEGF	837 [678–1033]	793 [592–1061]	882 [640–1214]	0.51	823 [617–1098]	851 [616–1175]	0.83

All units are pg/ml except *denoting ng/ml, data represented as Geometric mean [95% CI].

We further stratified the population by the sputum neutrophil count as we had previously reported relationships between CT parameters and neutrophilic inflammation. The clinical and CT imaging characteristics and sputum mediator concentrations for subjects stratified by differential sputum neutrophil count and the median RB1 total and luminal areas are as shown (Tables 4, 5, 6, 7). Subjects in the high neutrophil, high RB1 total or luminal area groups had significantly the highest number of severe exacerbations in the previous year and the highest proportion of subjects with qualitative CT evidence of bronchiectasis. Those in the low neutrophil and high RB1 total or luminal area groups had significantly the lowest body mass index (BMI). No other significant differences in clinical characteristics were observed (Tables 4 and 6).

**Table 4:** Clinical features of subjects stratified by the median neutrophil count (%) and RB1 total area

	**Low neutrophil, Low total area**	**High neutrophil, Low total area**	**Low neutrophil, High total area**	**High neutrophil, High total area**	**p value**
N	14	15	13	17	
Male, n	5	9	3	8	0.24
Current age	52 (3)	54 (3)	44 (4)	47 (3)	0.15
Age of onset	36 (5)	31 (5)	24 (5)	20 (4)	0.08
BMI, kg/m^2^	32.7 (1.6)	32 (2)	24.4 (1.4)	29.7 (0.8)	**0.002**
Habitual and ex smokers with >10 pack year history	4	5	2	2	0.95
Pack years history	6.5 (3)	14.5 (9)	7 (4)	5 (3)	0.59
Severe Exacerbations	1.5 (0.5)	2.5 (0.4)	2.5 (0.6)	4 (0.6)	**0.01**
IgE	208	221	270	119	0.28
ACQ_6_	2.2 (0.25)	2.2 (0.26)	1.9 (0.31)	2.3 (0.27)	0.68
Subjects taking oral corticosteroids, n	7	8	4	6	0.87
Daily Predinsolone dose, mg	6 (1.9)	5.5 (1.6)	3 (1.5)	2 (0.7)	0.14
Pre Bronchodilator FEV_1_	2.21 (0.3)	2.29 (0.2)	2.24 (0.2)	2.31 (0.25)	0.99
Post Bronchodilator FEV_1_	2.36 (0.3)	2.48 (0.2)	2.41 (0.2)	2.42 (0.25)	0.98
Bronchodilatorreversibility, mls	0.15 (0.03)	0.46 (0.2)	0.17 (0.06)	0.11 (0.04)	0.28
Pre Bronchodilator FVC	3.12 (0.3)	3.31 (0.2)	3.33 (0.2)	3.26 (0.32)	0.96
Post Bronchodilator FVC	3.24 (0.3)	3.49 (0.2)	3.39 (0.2)	3.28 (0.32)	0.92
Pre Bronchodilator FEV_1_ % of predicted	74.4 (8.2)	77.6 (5.6)	76.7 (4.9)	74.1	0.93
Post Bronchodilator FEV_1_ % of predicted	79.4 (7.8)	82.1 (6.2)	83.4 (4.3)	83.8	0.94
Bronchodilator Reversibility % FEV_1_	5.03 (1.1)	5.8 (1.3)	6.7 (1.9)	5.5 (1.6)	0.94
Pre Bronchodilator FEV_1_/FVC ratio	69.8 (4.1)	69.8 (3.5)	68 (3.3)	70.3 (4.1)	0.97
Post Bronchodilator BD FEV_1_/FVC ratio	72.1 (4)	70.7 (3.1)	72.9	73.2 (4)	0.96
Total Cells	0.53 [0.2-1.43]	0.7 [0.41-1.4]	0.31 [0.15-0.63]	0.82 [0.4-1.4]	0.79
Neutrophils %	35 [19.6-62.5]	86 [80.5-91.9]	39.5 [29.5-52.5]	83.8 [78.4-88.6]	**<0.001**
Eosinophils %	2.8 [1.1-7]	2.2 [0.9-9.3]	3.7 [1.4-10.8]	1.2 [0.2-2.7]	0.16
Bronchiectasis present n (%)	2 (14)	5 (33)	5 (38)	11 (65)	**0.03**
Bronchial Wall Thickening present	10 (70)	11 (73)	8 (62)	10 (63)	0.87
Lumen area/Body Surface Area (mm^2^/m^2^)	3.6 (0.26)	3.3 (0.35)	7.1 (0.66)	7.2 (0.58)	**<0.001**
Total area/Body Surface Area (mm^2^/m^2^)	13.3 (0.54)	12.1 (0.67)	21.8 (1.64)	23.6 (1.27)	**<0.001**

**Table 5:** Sputum mediator concentrations for subjects stratified by the median neutrophil count (%) and RB1 total area

	**Low neutrophil, Low total area**	**High neutrophil, Low total area**	**Low neutrophil, High total area**	**High neutrophil, High total area**	**p value**
Factor VII*	2.19 [1.59-3.01]	1.88 [1.5-2.37]	2.06 [1.51-2.83]	2.41 [1.89-3.07]	0.61
Fibrinogen*	271 [165–442]	249 [103–602]	94 [60–148]	430 [220–830]	**0.002**
Ferritin*	2.28 [1.06-4.88]	2.93 [1.63-5.26]	1.74 [0.99-3.07]	2.11 [0.79-5.66]	0.5
IL-1 α	38 [18–79]	43 [29–64]	13 [7–24]	28 [14–58]	**0.001**
IL-1 β	34.6 [17.4-69]	56.6 [23.7-135]	15.8 [8.87-28.2]	117 [47–290]	**0.005**
IL-1RA	8249 [3792–17942]	8053 [4281–15148]	4341 [2370–7952]	7599 [4441–13002]	0.39
IL-6	40.7 [20.1-82.6]	26.1 [12.3-55.5]	41 [23.3-72.3]	62.7 [35.1-112]	0.27
IL-8	3469 [1790–6726]	2365 [865–6646]	3857 [2088–7124]	5138 [2186–12076]	0.54
IL-15	390 [260–600]	430 [290–670]	410 [250–670]	500 [360–700]	0.43
IL-18	35 [22–57]	38 [23–64]	34.7 [22.6-53.5]	50.3 [30.8-82]	0.32
TNF-α	4.64 [3.47-6.2]	4.7 [2.21-9.99]	5.8 [3.74-9]	10.5 [4.67-23.7]	0.07
TNFR2	340 [140–830]	240 [100–560]	240 [130–460]	540 [230–1230]	0.54
CCL2	153 [92–256]	62.2 [31–124]	104 [53–205]	10 6[62–181]	0.079
CCL4	380 [225–640]	380 [165–877]	866 [299–2508]	414 [174–983]	0.096
CCL11	56 [39.5-79.4]	41.2 [32.3-52.4]	137 [72–261]	54.6 [36.1-82.5]	**0.0047**
MMP-3*	0.29 [0.14-0.57]	0.25 [0.14-0.42]	0.20 [0.11-0.36]	0.26 [0.16-0.43]	0.58
MMP-9*	41.3 [15.5-110]	55.7 [29.9-103]	11.6 [7.16-19]	65.8 [36–120]	0.17
TIMP-1*	153 [104–225]	58 [23–145]	139 [74–226]	114 [62–210]	0.25
ICAM-1	4050 [2250–7280]	1520 [930–2510]	4800 [2220–10600]	2600 [1570–4320]	**0.017**
VCAM-1	1400 [690–2820]	1080 [670–1750]	1020 [440–2350]	1040 [670–1630]	0.47
VEGF	1109 [799–1539]	579 [370–907]	903 [551–1481]	865 [542–1381]	0.061

All units are pg/ml except *denoting ng/ml, data represented as Geometric mean [95% CI].

**Table 6:** Clinical features of subjects stratified by the median neutrophil count (%) and RB1 luminal area

	**Low neutrophil, Low luminal area**	**High neutrophil, Low luminal area**	**Low neutrophil, High luminal area**	**High neutrophil, High luminal area**	**p value**
N	13	16	14	16	
Male	4	10	4	7	0.22
Current age	52 (3)	54 (3)	44 (3)	46 (3)	0.14
Age of onset	35 (4)	31 (4)	25 (4)	20 (4)	0.16
BMI, kg/m^2^	32.8 (1.7)	32.4 (1.8)	24.9 (1.4)	29.1 (0.8)	**<0.001**
Habitual smokers and ex smokers with >10 py	4	6	2	1	0.86
Pack years history	7 (3)	15 (8)	6.5 (4)	4 (3)	0.48
Severe Exacerbations	1.5	2	2	4	**0.05**
IgE	266	212	174	141	0.38
ACQ_6_	2.11 (0.24)	2.29 (0.24)	1.98 (0.30)	2.29 (0.28)	0.82
Subjects taking oral corticosteroids	7	7	4	7	0.84
Daily predinosolone dose, mg	6.9 (2)	3.9 (1.2)	3.0 (1.4)	3.4 (1.3)	0.30
Pre Bronchodilator FEV_1_	2.15 (0.32)	2.21 (0.2)	2.29 (0.19)	2.39 (0.25)	0.90
Pre Bronchodilator FEV_1_ % predicted	2.29 (0.31)	2.37 (0.22)	2.47 (0.2)	2.53 (0.24)	0.90
Bronchodilator reversibility, ml	0.14 (0.02)	0.42 (0.25)	0.18 (0.05)	0.14 (0.04)	0.44
Pre Bronchodilator FVC	3.07 (0.37)	3.18 (0.27)	3.37 (0.2)	3.40 (0.31)	0.84
Pre Bronchodilator FVC	3.17 (0.37)	3.36 (0.28)	3.44 (0.2)	3.41 (0.31)	0.92
Pre Bronchodilator FEV_1_ % of predicted	74.8 (8.9)	73.9 (5.7)	76.1 (4.5)	75.9 (5.3)	0.99
Post Bronchodilator FEV_1_ % of predicted	79.7 (8.4)	77.4 (6.2)	82.8 (4)	83.4 (4.9)	0.87
BD Bronchodilator Reversibility % FEV1	4.8 (1.1)	4.7 (1.1)	6.7 (1.8)	7.4 (2.8)	0.68
Pre Bronchodilator FEV_1_/FVC ratio	69.2 (4.4)	70 (3.3)	68.7 (3.2)	70.1 (4.3)	0.99
Post Bronchodilator FEV_1_/FVC ratio	71.4 (4.3)	70.1 (2.8)	72.9 (2.8)	74 (4.2)	0.87
Total Cells	0.45 [0.16-1.2]	0.80 [0.45-1.4]	0.37 [0.17-0.80]	0.8 [0.4-1.5]	0.74
Neutrophils %	33.6 [18–62.7]	85.2 [79–91.1]	40.7 [30.9-53.6]	83.9 [78.9-89.6]	**<0.001**
Eosinophils %	2.7 [1–7.4]	1.9 [0.7-4.5]	3.7 [1.5-9.3]	1.4 [0.6-3.2]	0.32
Bronchiectasis present	2 (15)	7 (44)	5 (36)	9 (60)	0.15
Bronchial Wall Thickening present	10 (77)	12 (75)	8 (57)	9 (60)	0.59
Lumen area/Body Surface Area (mm^2^/m^2^)	3.4 (0.2)	3.1 (0.2)	7.0 (0.6)	7.6 (0.4)	**<0.001**
Total area/Body Surface Area (mm^2^/m^2^)	13.1 (0.5)	13.31 (1.1)	21.3 (1.5)	23.1 (1.4)	**<0.001**

**Table 7:** Sputum mediator concentrations for subjects stratified by the median neutrophil count (%) and RB1 luminal area

	**Low neutrophil, Low luminal area**	**High neutrophil, Low luminal area**	**Low neutrophil, High luminal area**	**High neutrophil, High luminal area**	**p value**
Factor VII*	2.23 [1.58-3.16]	1.85 [1.51-2.26]	2.03 [1.53-2.69]	2.54 [1.95-3.31]	0.36
Fibrinogen*	270 [160–460]	306 [141–662]	101 [65–157]	363 [166–793]	**0.037**
Ferritin	2280 [990–5220]	2150 [1100–4200]	1780 [1050–2990]	2750 [950–7900]	0.6
IL-1 α	43 [20–90]	34 [19–61]	13 [7–23]	26 [12–54]	0.14
IL-1 β	34.3 [16.2-72.6]	57 [25.1-127]	17 [9.81-29.4]	121 [45.8-323]	**0.005**
IL-1RA	9021 [3972–20484]	9180 [5058–16662]	4183 [2386–7332]	6642 [3826–11532]	0.054
IL-6	40.5 [18.5-88.7]	28.5 [14.2-57.2]	41.2 [24.6-68.9]	65.3 [35.3-120]	0.20
IL-8	3567 [1739–7316]	2705 [1155–6335]	3730 [2114–6581]	4716 [1710–13009]	0.49
IL-15	400 [250–640]	420 [290–610]	409 [269–623]	520 [360–740]	0.54
IL-18	37.3 [22.5-61.9]	41.1 [23.9-70.6]	33.4 [22.5-49.8]	49.2 [30.8-78.7]	0.19
TNF-α	4.62 [3.32-6.43]	4.81 [2.55-9.05]	5.53 [4.2-7.28]	11.1 [4.58-27.1]	0.19
TNFR2	350 [130–920]	250 [120–510]	250 [140–440]	550 [210–1410]	0.59
CCL2	158 [91–275]	69.1 [37.4-127]	104 [56.3-194]	98.7 [52.7-184]	0.38
CCL4	357 [205–621]	343 [159–742]	861 [330–2245]	464 [181–1189]	0.10
CCL11	56.7 [38.8-83.1]	43 [33.8-54.6]	122 [65.9-226]	53.4 [34.2-83.6]	**0.013**
MMP-3*	0.30 [0.14-0.62]	0.24 [0.15-0.38]	0.20 [0.12-0.34]	0.27 [0.16-0.47]	0.49
MMP-9*	37 [13–106]	63.2 [40.1-99.5]	13.9 [7.73-25]	58.2 [27.3-124]	**0.0015**
TIMP-1*	163 [110–241]	65 [29–145]	132 [73.5-239]	107 [51.7-221]	0.14
ICAM-1	3960 [2100–7493]	1600 [1030–2490]	4890 [2400–9970]	2660 [1500–4720]	**0.017**
VCAM-1	1270 [617–2620]	1140 [734–1790]	1140 [510–2560]	990 [610–1580]	0.54
VEGF	1192 [871–1632]	609 [398–932]	857 [537–136]	845[512–1393]	0.14

All units are pg/ml except *denoting ng/ml, data represented as Geometric mean [95% CI].

**Figure 2:**
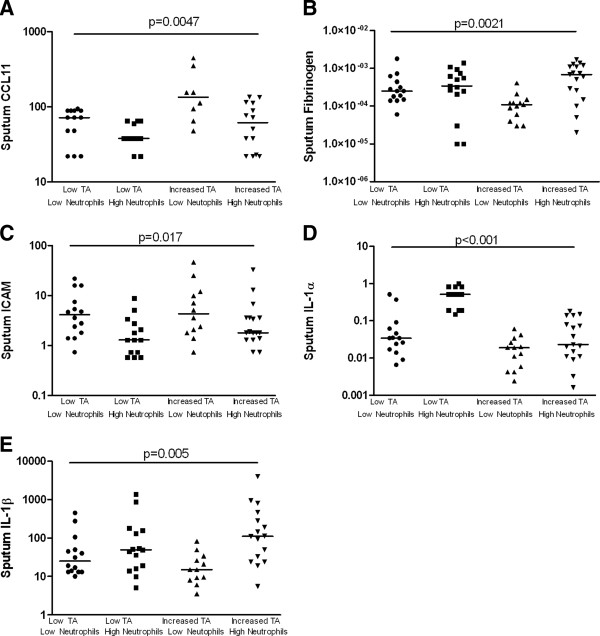
**Subjects stratified into four groups using the median sputum neutrophil count (≤66% versus >66%) and the RB1 total area (≤16 mm**^**2**^**/m**^**2 **^**versus >16 mm**^**2**^**/m**^**2**^**) on the X axis, their corresponding sputum levels of A) CCL11 (pg/ml), B) fibrinogen (ng/ml), C) ICAM (pg/ml) D) IL-1α (pg/ml) and e) IL-1β (pg/ml) on the log transformed Y axis.** Bar represents median value, p value by Kruskal Wallis ANOVA.

**Figure 3:**
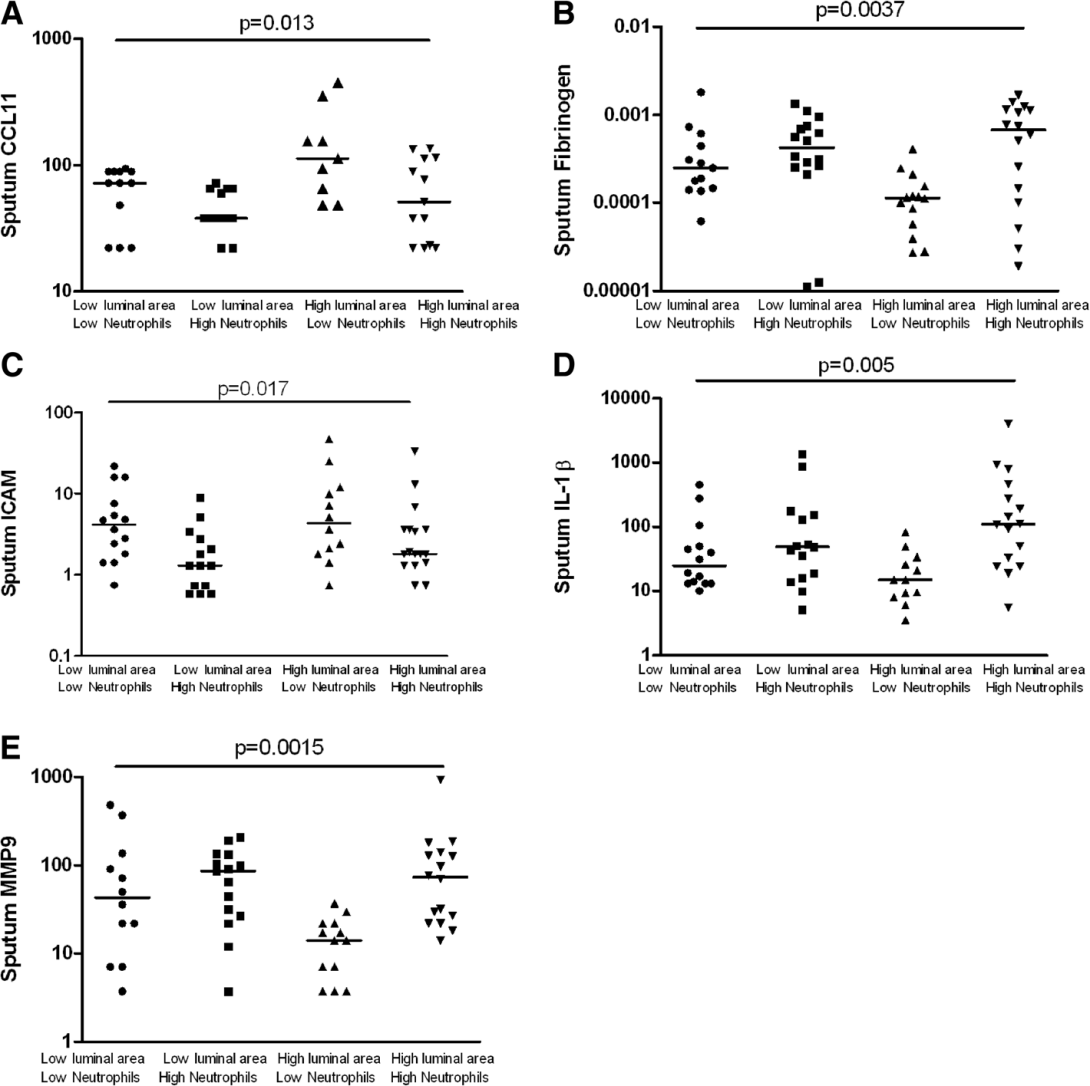
**Subjects stratified into four groups using the median sputum neutrophil count (≤66% versus >66%) and the RB1 luminal area (≤ 5 mm**^**2**^**/m**^**2 **^**versus >5 mm**^**2**^**/m**^**2**^**) on the X axis, their corresponding sputum levels of A) CCL11 (pg/ml), B) fibrinogen (ng/ml), C) ICAM (pg/ml) D) IL-1α (pg/ml) and E) MMP-9 (ng/ml) on the log transformed Y axis.** Bar represents median value, p value by Kruskal Wallis ANOVA.

The patterns in sputum mediator concentrations were very different between these groups. The sputum CCL11 concentration was elevated in those with a low neutrophil count and either a high RB1 total (p = 0.005) or luminal area (p = 0.004) (Figures 2 and 3). Reciprocally, these were the groups with the lowest sputum fibrinogen concentrations (p = 0.002 and p = 0.013 respectively). Similarly, sputum MMP9 was decreased in the low neutrophil group with high RB1 luminal area. Sputum ICAM was elevated in the groups with a low neutrophil count in both those with high or low RB1 total or luminal area whereas in contrast sputum IL-1β was elevated in those with a high neutrophil count irrespective of airway geometry. Sputum IL-1α was markedly increased in those with a high neutrophil count and a low RB1 total area (p < 0.001) (Figure 2).

Similar differences between groups were observed if patients were stratified by sputum eosinophil count (≤3% versus >3%) and median RB1 total and luminal areas with sputum CCL11 concentration greatest in those subjects with elevated sputum eosinophil count and high RB1 total or lumen area and in the same group the lowest sputum fibrinogen, and IL-1α concentrations (data not shown).

## Discussion

We report here differential sputum mediator profiles between groups of severe asthmatic patients stratified by airway wall geometry, with more marked differences between groups when patients were stratified further by sputum neutrophil counts. Some of the differences were closely related to the differential sputum cell counts such as sputum MMP9 and IL-1β, which were elevated in those groups with a high neutrophil count and sputum ICAM-1 was elevated in those subjects with a low sputum neutrophil count independent of airway geometry [27]. However, other differences were observed that were dependent upon both stratification by the sputum neutrophil count and airway geometry namely CCL11, IL-1α and fibrinogen. Sputum CCL11 concentration was elevated in subjects with a low sputum neutrophil count and high luminal and total area, whereas sputum IL1α was increased in subjects with a high sputum neutrophil count and low total area. Sputum fibrinogen was elevated in those subjects with luminal narrowing independent of sputum cellular differential, but was also increased in subjects with neutrophilic inflammation without luminal narrowing. These findings support the view that there are differential sputum mediator profiles related to airway geometry.

Several sputum mediators were associated more with neutrophilic inflammation than the pattern of airway geometry. Two of these mediators IL-1β and MMP-9 are observed in subjects with COPD and are reported to be associated with airway bacterial colonisation and neutrophilic inflammation. Interestingly, Matsumoto *et al.* observed an inverse relationship between sputum MMP-9 and%WA which was consistent with our observation of a low MMP-9 in the high luminal area, non-neutrophilic group. In contrast, sputum MMP9 was elevated in our study in those with neutrophilic inflammation independent of luminal area [28]. IL-1β and MMP-9 promote subepithelial fibrosis, subepithelial collagen deposition and elastin fibre disruption in asthma and in COPD are associated with many of the histopathological features of emphysema [29-32]. This may suggest a phenotype of remodelling associated more with ‘scaffold destruction’ leading to airway dilatation from loss of airway wall integrity as opposed to a predominant airway thickening. Indeed, the group with high total and luminal area were associated with increased qualitative evidence of bronchiectasis and frequent exacerbations. Although this process has relevance to those subjects with luminal dilatation, these mediators were also increased in subjects with neutrophilic inflammation and luminal narrowing. This suggests either that in these subjects an alternative inflammatory process is driving the airway narrowing or that there were changes that occurred earlier in the lung development of these subjects that led to either airway narrowing in early development or a predisposition to airway narrowing in the natural history of the disease.

Fibrinogen was also elevated predominately in those with neutrophilic inflammation, but was increased in subjects without neutrophilic inflammation with luminal narrowing and low RB1 total area. Fibrinogen is an acute phase soluble plasma glycoprotein, synthesised primarily in the liver and converted by thrombin into fibrin during blood coagulation. It is emerging as a promising systemic biomarker in COPD that shows relationships with disease severity, progression and mortality [33]. There are studies that link its presence to microvascular leakage from plasma exudation, which occurs during remodelling or neurogenic inflammation in asthma [34,35] and may contribute to pathophysiological features of airway hyperresponsiveness [36]. Fibrinogen leakage exudes into the extravascular compartment and interacts with neutrophils causing their sequential activation and promoting their survival by reducing apoptosis [37]. Sputum fibrinogen is therefore likely to be increased in subjects with airway wall oedema, which may contribute to the luminal narrowing in subjects independent of sputum cell differential. Interestingly, it was not increased in subjects without a sputum neutrophilia and without high RB1 suggesting that in this group airway wall oedema and increased vascularity may not play a prominent role. This needs to be confirmed by examination of the bronchial wall in this group of subjects. Intriguingly, this group also had elevated sputum CCL11 and the highest sputum eosinophil count, albeit non-significant, suggesting that although eosinophilic inflammation may be important in driving remodelling and luminal narrowing in some subjects it is also associated with normal airway geometry. Whether this represents a group of patients protected from airway remodelling or at an earlier stage of the natural history needs to be further explored.

In contrast to sputum IL-1β, sputum IL1α was not closely related to neutrophilic inflammation but was increased in those subjects with low RB1 luminal area increased and in those subjects with both low RB1 total area and neutrophilic inflammation. Recent evidence has implicated IL-1α as an important link between the innate immune system and allergic sensitisation TLR4 activation induces IL-1α release from epithelial cells, which acts in an autocrine manner to release GM-CSF and IL-33 [38]. IL-1α is a potent activator of fibroblasts [39] and might play a role in fibroblast activation and hyperplasia in asthma. In atherosclerosis [40] IL-1α has also been implicated in the plaque stability and outward vessel remodelling. Whether IL-1α plays a similarly important role in differential changes in airway geometry warrants further study.

One major criticism of our study is the cross-sectional design, which limits our interpretation of the dynamic relationship between the mediator profiles and airway remodelling. For example, it is uncertain whether the changes observed by CT imaging reflect progressive remodelling over many years as a consequence of airway inflammation or represent changes in airway geometry that were established in early lung development [41]. For example, there were small differences in the age of onset of disease and current age, albeit not disease duration, in subjects in the CT defined subgroups. This effect of time is particularly important when considering differential mediator profiles between subjects in whom the airway wall has remodelled towards the airway lumen versus those that have either maintained normal luminal patency or have remodelled away from the lumen and have airway dilatation. To address this question either longitudinal studies of the natural history of remodelling are required or studies of interventions that might impact in the short term upon remodelling such as bronchial thermoplasty [42,43]. Importantly, luminal area in the severe asthma group is decreased compared to controls such that even the high luminal area group described here do not represent bronchial dilatation compared to health. A further criticism is that sputum analysis reflects a composite measure of the proximal airways rather than a specific lobe whereas the CT imaging here focussed upon the dimensions of the RB1. Although a more comprehensive volumetric analyses the entire proximal airway tree would be informative we and others have reported that the RB1 geometry has good correlations with other proximal airways [44]. Therefore, we are confident that this is not a major limitation of this study. The sputum samples were processed with the mucolytic DTT, which affects the recovery of a number of mediators in particular Th2 cytokines. The scope of the mediators measured here are therefore hampered by this shortcoming, but none the less we have measured a comprehensive panel of biomarkers and are confident this reflects many aspects of the inflammatory profile in the airway of these subjects. Sputum analysis is restricted to sampling to the airway lumen and does not necessarily reflect airway inflammation in the airway wall. Large studies of the relationship between airway wall remodelling determined by bronchial biopsies and CT imaging are underway and the findings are eagerly awaited.

In conclusion, severe asthma is a complex heterogeneous disease and the interactions between airway inflammation and remodelling are poorly understood. Here we have demonstrated that sputum mediator profiling reveals a number of associations between airway geometry and sputum differential cell counts and mediator concentrations. Whether these findings reflect specific and consistent underlying biological phenotypes with predictable natural histories and the potential for stratified medicine approaches needs to be further investigated.

## Competing interests

DD, SG, SS, AS, WM, JE report no conflicts of interest. SV, HP and RK were employees of HoffmanLaRoche. CEB is on the advisory board for GlaxoSmithKline, AstraZeneca, HoffmanLaRoche, Novartis, Genentech and MedImmune. He has received consultancy fees from Medimmune and Novartis and travel expenses to attend scientific meetings from Boerhinger-Ingelheim.

## Authors’ contributions

DD, SG, SS and AS were involved in the recruitment of volunteers and in data collection. SG, SS, JE were involved with the qualitative and quantitative computerised tomography analysis. WM undertook the sputum cell count and sputum processing. SV, HP and RK were involved with arranging the mediator analysis. DD, SG, and CEB were involved in statistical analyses and interpretation. DD, SG, SS, RK, CB were involved in the design of the study and interpretation. DD, SG, RK and CEB were involved in the study design, volunteer recruitment, data collection, data interpretation, data analysis and had full access to the data and are responsible for the integrity of the data and final decision to submit. All authors contributed to the writing of the manuscript and have approved the final version for submission.
